# Partial wrap-clipping of the entrance of the pseudolumen of a fusiform aneurysm in the posterior inferior cerebellar artery: a technical note

**DOI:** 10.1007/s00701-017-3099-y

**Published:** 2017-01-31

**Authors:** Yasushi Motoyama, Yoshiaki Takamura, Shuichi Yamada, Young-Su Park, Hiroyuki Nakase

**Affiliations:** Department of Neurosurgery, Nara Medical University, 840 Shijo-cho, Kashihara, Nara 634-8522 Japan

**Keywords:** Partial wrap-clipping technique, Dissecting aneurysm, Fusiform aneurysm, Indocyanine green videoangiography

## Abstract

**Background:**

Fusiform aneurysms in the posterior inferior cerebellar artery (PICA) are rare and challenging to treat. Surgical treatment options for a fusiform aneurysm in the PICA include trapping with/without bypass and wrap-clipping, when elimination of the pathological wall from the systemic circulation and prevention of perforator injury are important. In addition, lower cranial nerve impairment due to surgical manipulation should also be avoided.

**Method:**

A fusiform-shaped aneurysm was found in a proximal part of the PICA by magnetic resonance angiography undertaken for evaluation of repeated vertigo in a 36-year-old man. The patient underwent direct surgery via a lateral suboccipital transcondylar fossa approach. The entrance of the pseudolumen was the only part to be wrapped and obstructed by clip application, through the corridor between the acoustic and glossopharyngeal nerves to avoid lower cranial nerve injury.

**Results:**

Indocyanine green (ICG) videoangiography demonstrated obliteration of pseudolumen and patency of peripheral PICA and perforator contributing to the medulla oblongata. The postoperative course was uneventful without periprocedural complications, including dysphagia and hoarseness.

**Conclusions:**

Partial wrap-clipping technique for obstruction of the entrance into a pseudolumen is one of alternatives for dissecting fusiform-shaped aneurysm in the PICA. ICG videoangiography was helpful to confirm the obliteration of the pseudolumen and patency of parent vessel and perforators.

## Introduction

Aneurysms located in the peripheral posterior inferior cerebellar artery (PICA) are rare and account for less than 1% of all intracranial aneurysms [6]. Fusiform-shaped aneurysm in the peripheral PICA has been reported previously, even though it is extremely rare and challenging to treat [4]. Some fusiform aneurysms have a dissecting nature and have also been described to transform to giant thrombosed or serpentine aneurysms in their future having a poor prognosis. Trapping or resection with/without vascular reconstruction for peripheral PICA contribution is a reasonable alternative from the point of view of elimination of the pathological wall. However, the proximal part of the PICA to the televelotonsilar segment has perforators contributing to the medulla oblongata that might affect severe ischemic complication, leading to poor prognosis if they had been sacrificed. The wrap-clipping technique is another surgical option for fusiform aneurysms with a dissecting nature, which have the advantage of patency in antegrade normal flow. Additionally, the location of the proximal PICA should be paid attention to considering lower cranial nerve injury while planning surgical treatment.

Herein we present the case of a dissecting fusiform aneurysm in the PICA with tendency for growing. In this case, only the entrance of the pseudolumen separated by the intimal flap was obstructed with the partial wrap-clipping technique in order to avoid lower cranial nerve injury. Indocyanine green (ICG) videoangiography was helpful to confirm the obliteration of the pseudolumen and patency of the parent vessel and perforators.

## Case report

**Fig. 1:**
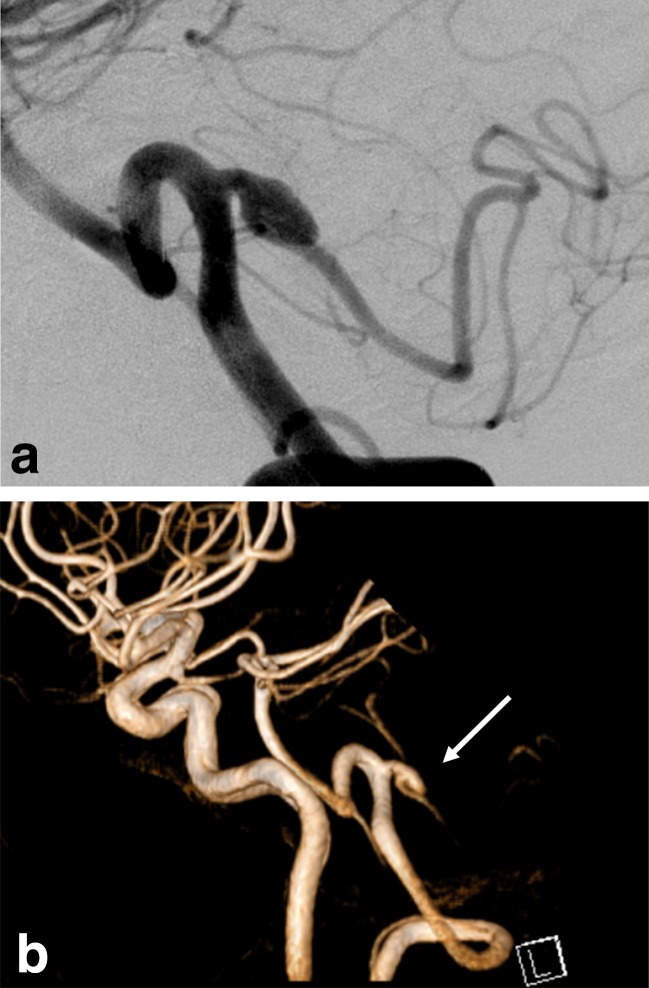
**a** Left vertebral artery (VA) angiography showing the aneurysm projecting along the parent PICA. **b** Three-dimensional angiography demonstrating the aneurysm originating not from the bifurcation with VA but from the proximal part of the PICA, mimicking a congenital berry aneurysm

**Fig. 2:**
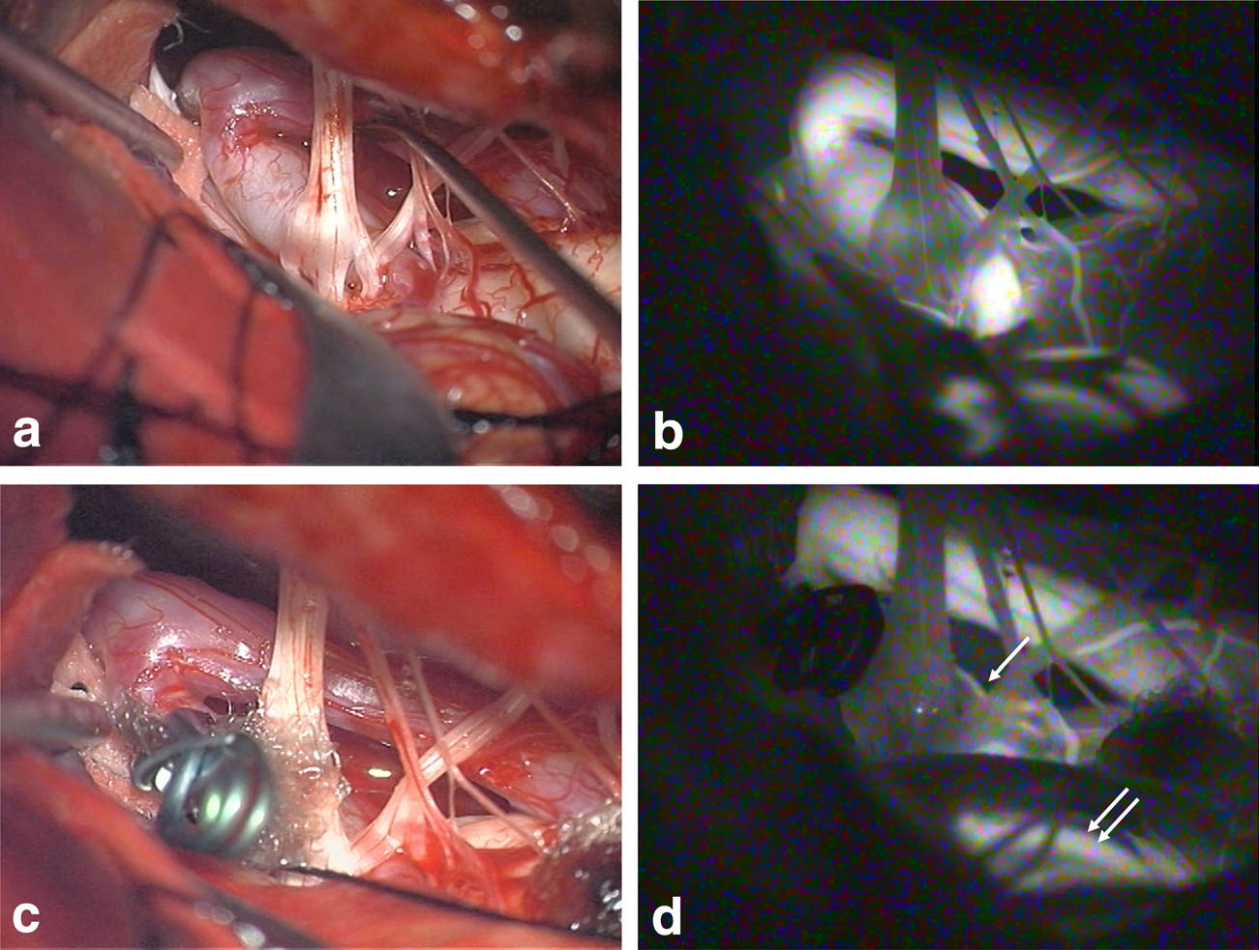
**a** Intraoperative view demonstrated the aneurysm to have a fusiform shape with thickened wall accompanied by a small amount of vaso vasorum. **b** Only the proximal part of the lateral wall of the dome was obliterated with right-angle clip application on wrapping with a sheet of polyglycolic acid (PGA). **c** Intraoperative indocyanine green (ICG) videoangiography demonstrated the aneurysm had a fusiform shape in the proximal PICA behind the lower cranial nerves. **d** ICG videoangiography after wrap-clip application revealed that the pseudolumen of the fusiform aneurysm was not illuminated but the peripheral PICA and perforating artery contributing to the lateral part of medulla oblongata were depicted

**Fig. 3:**
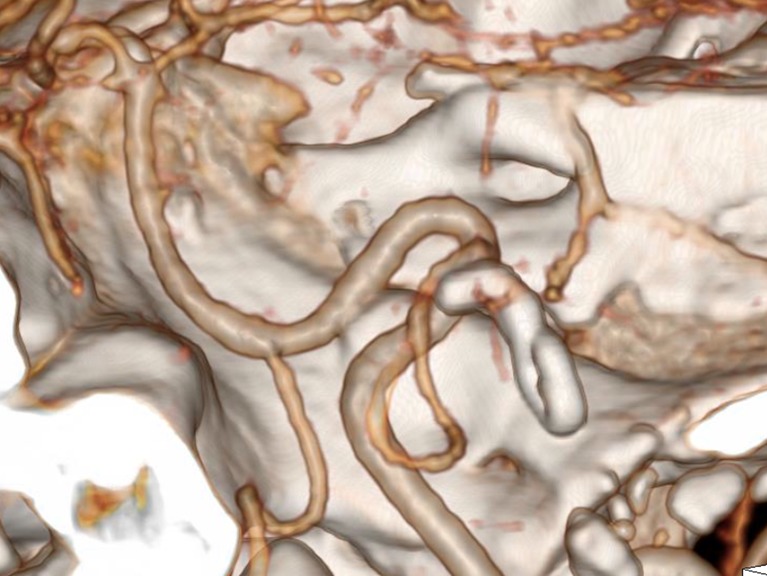
Postoperative 3DCTA demonstrating the aneurysm had disappeared and the PICA was patent

A 36-year-old man presented with a 1-year history of repeated vertigo, when magnetic resonance angiography (MRA) demonstrated an aneurysm originating from the proximal part of right PICA. Follow-up MRA showed an increase in size of the aneurysm after 6 months. Besides MRA, digital subtraction angiography and three-dimensional computed tomographic angiography (3DCTA) indicated that the aneurysm appeared to have an ellipsoid shape with an 8-mm maximum diameter and a distinct neck, and was diagnosed as a congenital berry aneurysm (Fig. 1). In consideration of his young age and the size of the aneurysm, we decided to perform some intervention for prevention of future subarachnoid hemorrhage (SAH). The patient underwent right lateral suboccipital craniotomy via transcondylar fossa approach in the park bench position. The aneurysm was revealed to be located just after in the proximal part of PICA and have a fusiform shape with a thickened wall accompanied by a small amount of vaso-vasorum. The aneurysm did not have any neck and dome separated from the parent artery and was accompanied by perforating artery contributing to the lateral surface of medullar oblongata observed behind the aneurysm itself (Fig.2a), which was also confirmed on ICG videoangiography (Fig.2b). For avoidance of lower cranial nerve injury and perforating arterial injury that would lead to the Wallenberg’s syndrome, if the aneurysm was obliterated by trapping or proximal clipping, we tried to occlude only the pseudolumen with clipping. The aneurysm was dissected in the entire circumference from the medulla oblongata. A sheet of polyglycolic acid (PGA) was used to wrap the aneurysm with the parent artery and perforating artery. A right-angle-shaped clip with the appropriate length of blade was applied at the upper surface of the proximal part of the fusiform aneurysm over the PGA sheet with the aim of obstructing the entrance of the pseudolumen in the space between the acoustic and glossopharyngeal nerves (Fig.2c). ICG videoangiography demonstrated no evidence of occlusion or delay in flow of the peripheral part of the PICA and the perforating artery contributing to the lateral part of medulla oblongata. However, the fusiform aneurysm was no longer illuminated on the ICG videoangiography, which indicated that the entrance of the pseudolumen had been occluded (Fig.2d). The postoperative course was uneventful without any neurological deficits, including dysphagia and hoarseness. Three-dimensional CTA demonstrated complete occlusion of the pseudolumen of the fusiform aneurysm with patency of the PICA (Fig. 3). Follow-up MRA did not demonstrate any evidence of recurrence 1 year after operation.

## Discussion

Proximal ligation, trapping, and resection have been reported as curative treatment options for intracranial arterial dissecting or fusiform aneurysms, as well as endovascular internal trapping. Even though these treatments are reasonable for the concept of elimination of pathological vascular walls from the systemic flow, normal blood flow would be consequently sacrificed to require vascular reconstruction. Even if that were the case, the danger of perforating arterial injury would remain.

The wrap-clipping method has been established as one of treatment options for arterial dissection and blood blister aneurysm (BBA), which can provide reinforcement of holding the applied clip to result in prevention of re-rupture and enlargement of the pathological wall [1–3]. This method has been reported to be especially useful for BBA in the distal ICA and dissecting aneurysm for the PICA, having the advantage of preserving antegrade blood flow of the parent artery and perforating arteries originating from that part of the affected vessel [1, 3]. In our patient, preoperative imaging demonstrated the location of a pseudolumen of a fusiform aneurysm was lateral to the parent artery and the entrance of the PICA between acoustic nerve and glossopharyngeal nerve. Additionally, the fusiform aneurysm was in the proximal PICA, just behind the lower cranial nerves under intraoperative inspection. In this type of aneurysm surgical intervention has been frequently implicated with lower cranial nerve dysfunction postoperatively [9]. Therefore, in order to avoid lower cranial nerve injury we applied this wrap clip technique in a limited area between those cranial nerves.

A variety of materials have been reported for wrapping, including muscle fascia, cotton, Goatex, and Dacron [2, 3]. For wrapping, the material require durability to prevent rupture of the pathological wall without elimination from circulation and moldability to preserve the perforators and to protect fragile surrounding tissue like lower cranial nerves. We used PGA as a material for wrapping of the artery in this operation. PGA is soft and easy to handle and tough enough to hold the artery tight with a clip. Furthermore, PGA as a material for wrapping has an effect on healing of vasculature, although this was investigated in experiments using animal models [4]. However, it is hydrolyzed and absorbed in 3 weeks. Besides durability and moldability, longevity should also be considered for a wrapping material, in order to prevent future growth of the dissecting aneurysm. Some types of fusiform aneurysm or dissecting aneurysm have been reported to have growth potential, especially in partially thrombosed aneurysms. In such cases, even trapping could not prevent the aneurysm from growing [5]. Fortunately, in our case 1-year later follow-up magnetic resonance imaging (MRI) demonstrated no evidence of recurrence of the aneurysm, even though further follow-up is needed. PGA would be enough for the fusiform aneurysm due to dissection in an early stage to cover the pathological wall appropriately. In consideration of long-term prognosis, however, especially in the case of fusiform aneurysm with a partially thrombosed part which has been reported to have growth potential, it might be better to opt for wrapping of the artery using other types of material which have durability in the long-term, such as Goatex [2].

A dissecting aneurysm occasionally has a fusiform shape besides a tapered occlusion and pearl and string signs. On cerebral angiography, some fusiform aneurysms show the intimal flap itself, like in our case, as well as a double lumen, pooling, and/or slow filling along the vessel, as an evidence of dissection. However, many of them do not reveal any apparent findings supporting a dissecting nature. For precise diagnosis of fusiform aneurysm, not only catheter angiography and 3DCTA that mainly provide information regarding the intra-vascular space but also MRI are important for depicting the character of the wall and thrombus inside the aneurysm. Basi-parallel anatomical scanning (BPAS) MRI has been reported to be useful for diagnosis of vertebral artery dissection [8]. MRI-BPAS can depict the outer appearance of the vasculature to make a precise diagnosis of dissection while simultaneously observing the internal space by MRA or cerebral angiography. In our case, more detailed MRI could make correct diagnosis of a fusiform-shaped aneurysm before operation.

**Fig. 4:**
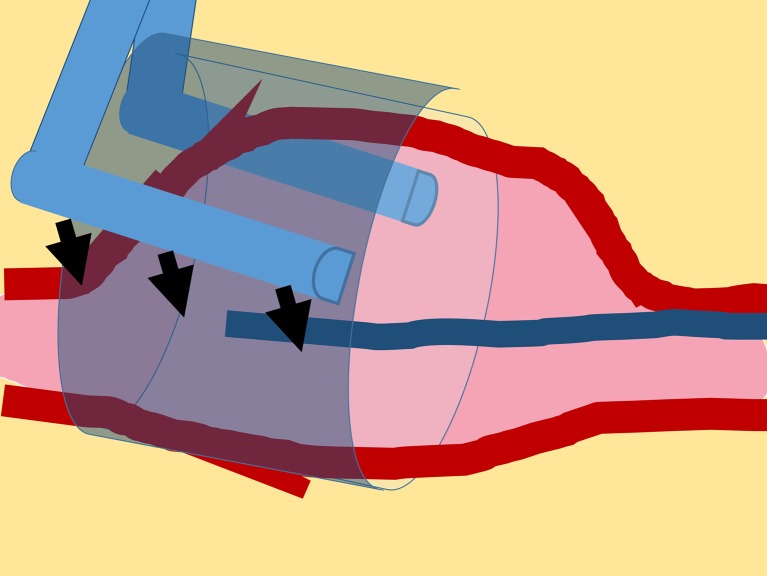
Schematic drawing illustrated the right-angle clip over the wrapping, obliterating only the entrance of pseudolumen

In this paper, only the entrance of the pseudolumen was obliterated with partial clip application (Fig. 4). Meticulous inspection and careful maneuvering around the surrounding fragile nervous structures showed the aneurysm to be dissected entirely. Preoperative neuro-imaging could inform the surgeons of the location and size of the entrance of pseudolumen from its external appearance. However, preservation of the parent vessel and perforators and obliteration of the pseudolumen could not be visualized with direct microscopic inspection, but was clearly confirmed by ICG videoangiography, which was extremely useful. ICG videoangiography is considered a useful tool for intraoperative control of aneurysm clipping, bypass surgery, and surgical occlusion of arteriovenous fistulas [7]. This technique generates real-time information regarding vessel flow, even in tiny perforators that are otherwise difficult to identify with digital subtraction angiography or 3DCTA, and in the situation of SAH.

## Conclusion

The wrap-clip technique for obliteration of only the entrance into a pseudolumen might be one of the alternatives for treating a dissecting fusiform-shaped aneurysm, which was effective for avoidance of lower cranial nerve injury in the case of the proximal part of the PICA. ICG videoangiography played an extremely important role in confirmation of obliteration of the pseudolumen and patency of the parent vessel and perforators.
